# Supplementary material for: Absence of Grail promotes CD8+ T cell anti-tumour activity
Source: Nat Commun. 2017 Aug 10;8:239. doi: 10.1038/s41467-017-00252-w (PMC5552797; doi:10.1038/s41467-017-00252-w)
Supplement: Supplementary file 1 — Supplementary Information [file 41467_2017_252_MOESM1_ESM.pdf]

File Name: Supplementary Information
Description: Supplementary Figures

File Name: Peer Review File
Description:

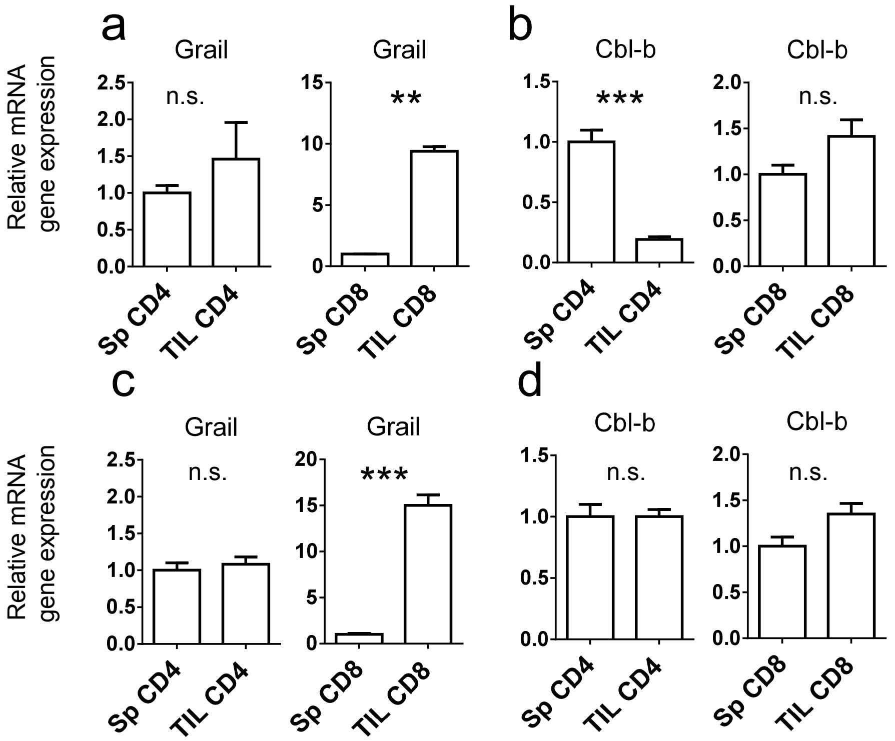

Supplementary Figure 1: Different expression pattern of Grail and Cbl-b in CD4$^+$ and CD8$^+$ T cells from spleen and TILs. EG-7 (**a,b**) or EL-4 cells (**c,d**) were injected subcutaneously into the flanks of wild-type (WT) mice and 17 days later WT CD4$^+$ and CD8$^+$ T cells from spleens and TILs were FACS-sorted and restimulated with plate-bound anti-CD3 for 4 hours. The mRNA expression of Grail (**a,c**) and Cbl-b (**b,d**) was detected by real time (RT)-PCR analysis. Results for target genes are presented after normalizing to β-actin and shown as mean ± sem. All experiments were independently performed twice with 10 mice per group. ** $p < 0.01$, *** $p < 0.001$, n.s.: not significant as determined using a Student's t test.

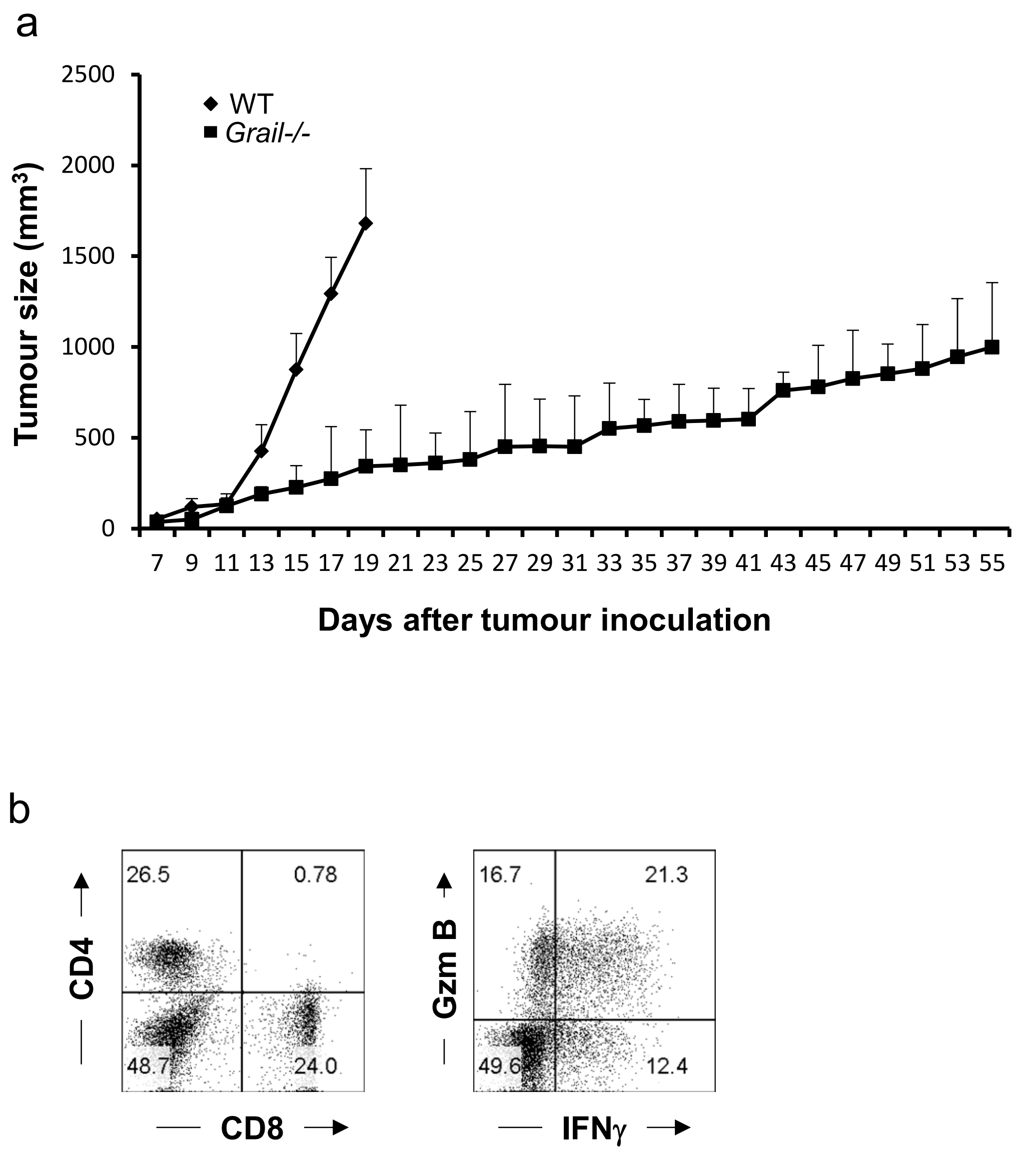

Supplementary Figure 2: Long-term tumour control is mediated by Grail absence. WT and *Grail-/-* mice were inoculated with EG-7 tumours and monitored as described in Fig. 1a. WT mice were euthanized at day 17 due to tumour size and *Grail-/-* mice were monitored until day 55. Tumour burden is shown in (**a**). The frequency of $CD4^+$ and $CD8^+$ TIL subsets from *Grail-/-* mice at day 55 as well as GrzB and IFNγ expression by $CD8^+$ TIL is shown in (**b**). (n=10 mice per group).

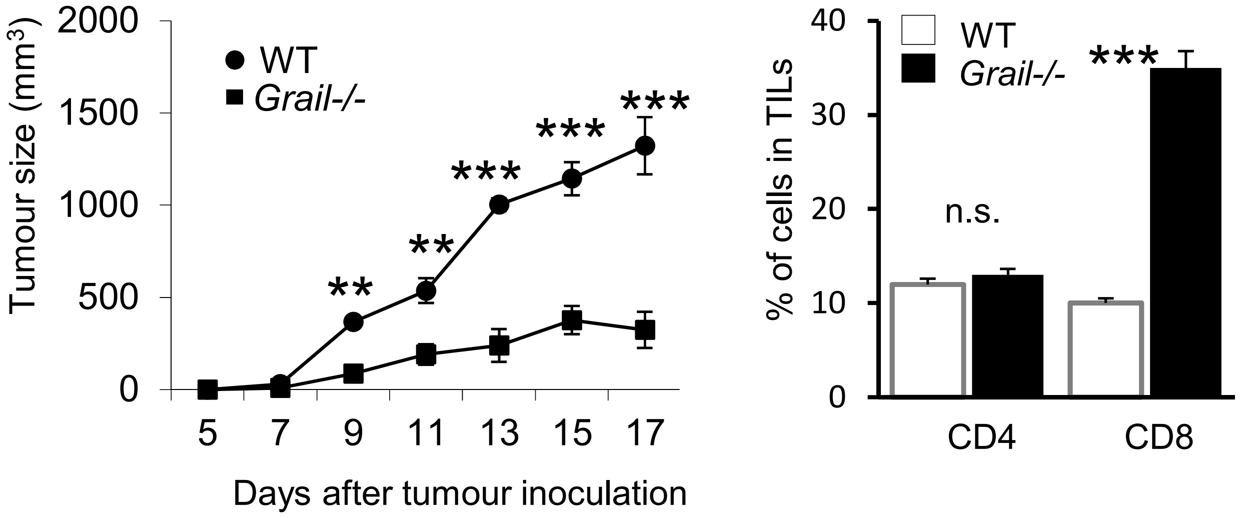

Supplementary Figure 3: EL-4 lymphoma growth is controlled in *Grail-/-* mice. WT and *Grail-/-* mice were injected with EL-4 tumour cells subcutaneously on day 0, and from day 7 tumour growth was monitored and calculated as in Fig.1a (left graph). Tumour infiltrating lymphocytes (TILs) isolated from EL-4 tumours were stained with anti-CD4 and CD8α antibodies (right bar graph). The graph shows mean ± sem. The results shown are representative of three independent experiments with 5-8 mice per group. ** $p < 0.01$, *** $p < 0.0001$, n.s.: not significant as determined using a Student's t test.

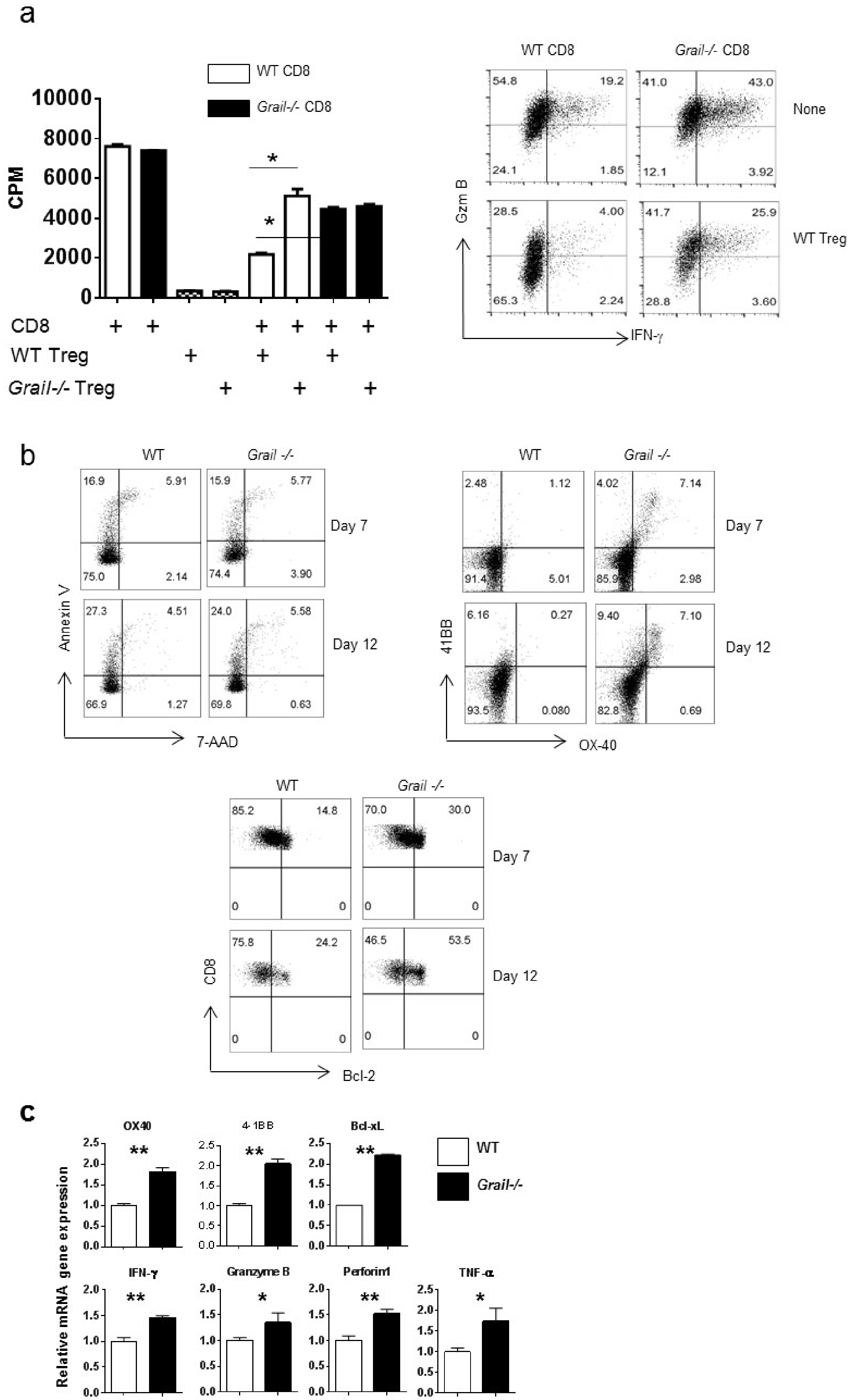
a
WT CD8
Grail-/- CD8
10000
8000
6000
4000
2000
0
CPM
*
*
CD8
WT Treg
Grail-/- Treg
WT CD8
Grail-/- CD8
54.8
19.2
24.1
1.85
41.0
43.0
12.1
3.92
None
28.5
4.00
65.3
2.24
41.7
25.9
28.8
3.60
WT Treg
Gzm B
IFN-γ
b
WT
Grail -/-
16.9
5.91
75.0
2.14
15.9
5.77
74.4
3.90
Day 7
27.3
4.51
66.9
1.27
24.0
5.58
69.8
0.63
Day 12
Annexin V
7-AAD
WT
Grail -/-
2.48
1.12
91.4
5.01
4.02
7.14
85.9
2.98
Day 7
6.16
0.27
93.5
0.080
9.40
7.10
82.8
0.69
Day 12
41BB
OX-40
WT
Grail -/-
85.2
14.8
0
0
70.0
30.0
0
0
Day 7
75.8
24.2
0
0
46.5
53.5
0
0
Day 12
CD8
Bcl-2
c
OX40
4-1BB
Bcl-xL
WT
Grail-/-
IFN-γ
Granzyme B
Perforin1
TNF-α
Relative mRNA gene expression
**
**
**
**
*
**
*

Supplementary Figure 4: Resistance to Treg suppression in addition to increased activation and functionality of $CD8^+$ T cells in the absence of Grail. (**a**) Naïve WT or *Grail-/-* $CD8^+$ T cells were cultured with or without FACS-sorted WT or *Grail-/-* Foxp3-GFP^+CD4^+ nTreg cells in triplicate wells in the presence of plate-bound anti-CD3 and irradiated WT APCs. Proliferation was assayed 72 hrs after treatment by adding [^{3}H]-thymidine to the culture for the last 8 hrs. 2 days later, expression of IFN-γ and granzyme B was analyzed by intracellular staining. Numbers in dot plot quadrants represent the percentages. (**b**) WT and *Grail-/-* TIL were isolated from EG-7 tumours on days 7 and 12 and stained with anti-CD8α, 41BB, OX40, Bcl2, Annexin V antibodies and 7AAD. Numbers in each dot plot indicate the percentage of cells within each quadrant from a representative tumour at each time point and is sub-gated on $CD8^+$ T cells. The results shown are representative of two independent experiments with 5 mice per group. (**c**) RT-PCR analysis of mRNA levels of indicated genes in FACS-sorted WT and G*rail-/-* $CD8^+$ T cells from tumours on day 17 following tumour inoculation. Results for target genes are presented after normalizing to β-actin as mean ± sem for both groups from 3 independent experiments. * $p < 0.05$, ** $p < 0.01$ as determined using a Student's t test.

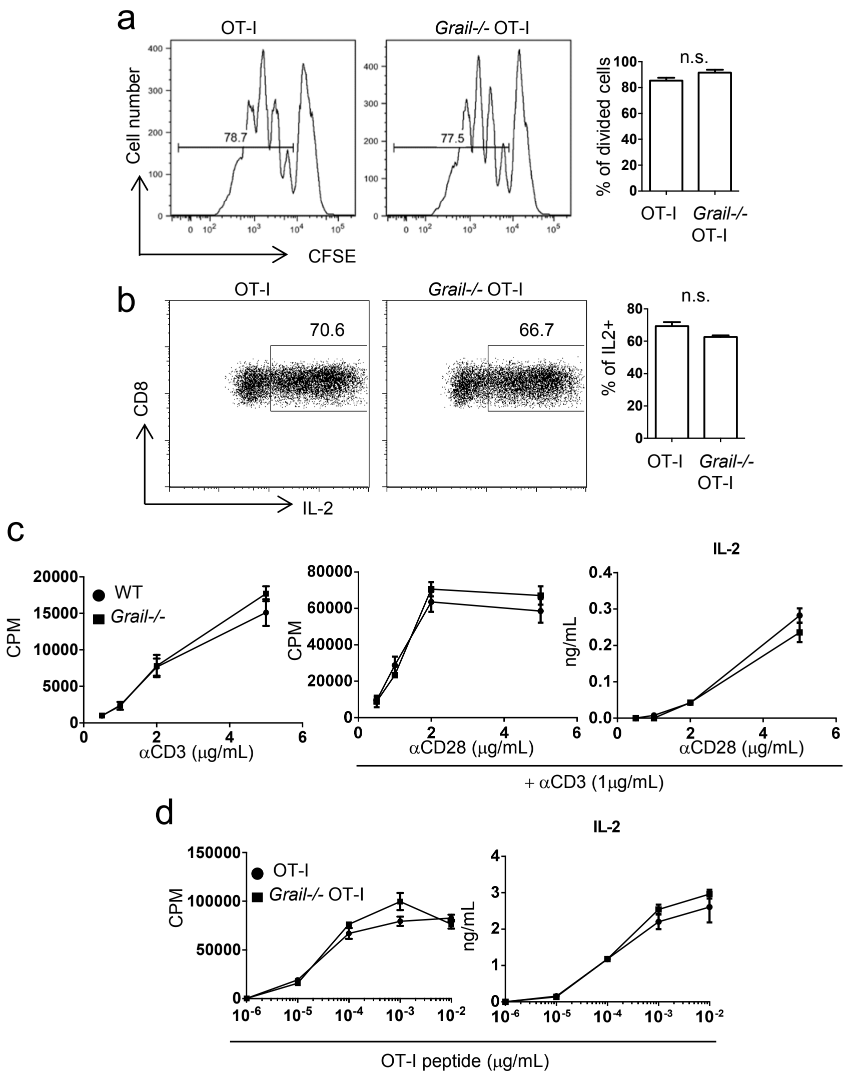

Supplementary Figure 5: $CD8^+$ T cell priming in the absence of Grail. (**a-b**) Purified $CD45.2^+$ OT-I and *Grail-/-* OT-I cells were labeled with CFSE and transferred into $CD45.1^+$ congenic mice (3 mice per group) which were injected with EG-7 tumour cells 5 days earlier. 3 days later mice were sacrificed and the draining lymph node cells (DLNs) were analyzed. (**a**) Percentage of divided donor cells ($CD45.2^+CD8^+$). (**b**) DLN cells were stimulated for 5 hrs with OVA peptide and IL-2 production was analyzed in the $CD45.2^+CD8^+$ gate by intracellular staining. Numbers in dot plot quadrants represent the percentages. (**c**) FACS-sorted naïve $CD8^+$ T cells were activated with plate-bound anti-CD3 alone or together with anti-CD28. Proliferation was assayed by adding [^{3}H]-thymidine to the culture for the last 8 hours of the 72-hour culture. IL-2 production was measured by ELISA using supernatants collected 24 hr after activation. (**d**) Naïve OT-I and *Grail-/-* OT-I cells were activated with OVA peptide and irradiated WT APCs. Proliferation was assayed by adding [^{3}H]-thymidine to the culture for the last 8 hours of the 48-hour culture. IL-2 production was measured by ELISA using supernatants collected 24 hr after activation. The bar graph shows mean ± sem. All experiments were independently performed twice. n.s.: not significant as determined using a Student's t test.

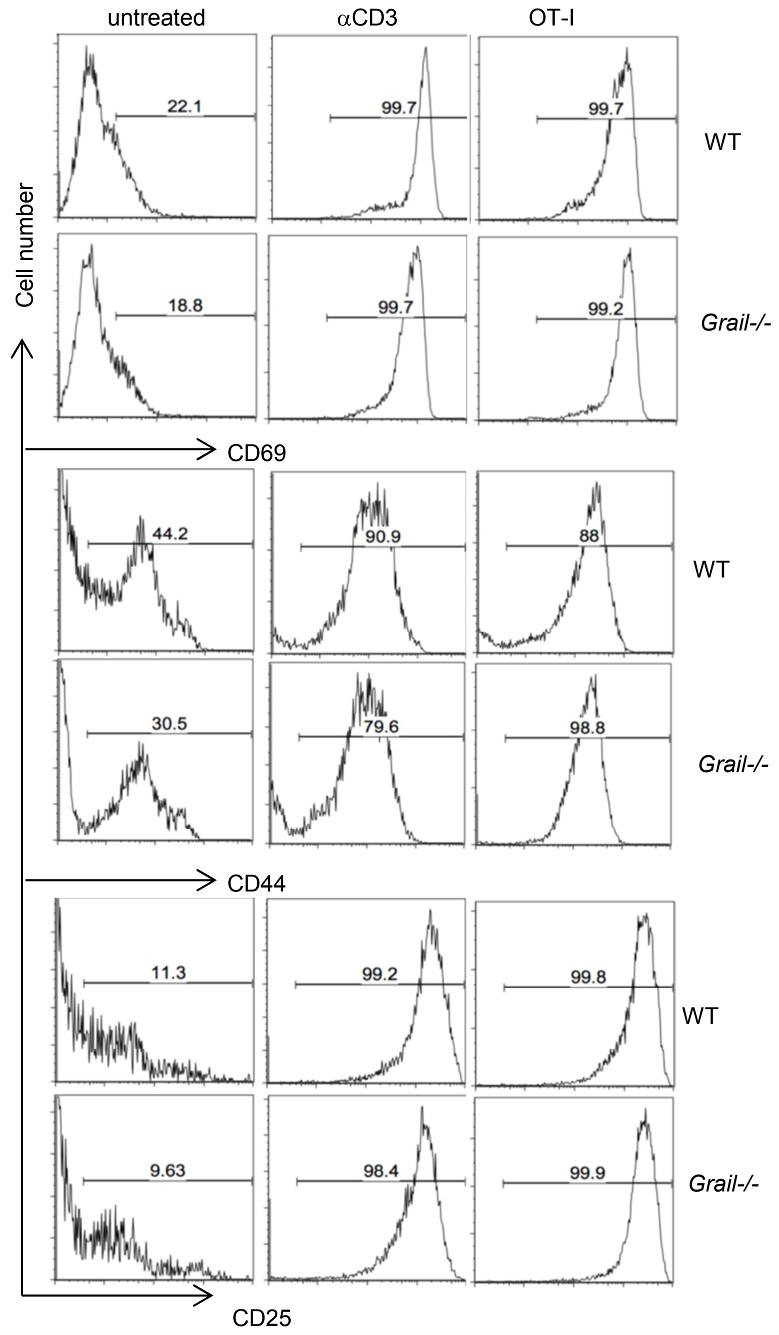

Supplementary Figure 6: The activation of CD8^{+} T cells *in vitro*. WT and *Grail-/-* splenocytes were left untreated or activated in the indicated conditions for 24 hours, followed by surface staining of CD8, CD69, CD44 and CD25. Expression was analyzed in the CD8^{+} gate. The gates and numbers indicate the percentage of positive cells. All experiments were independently performed twice.

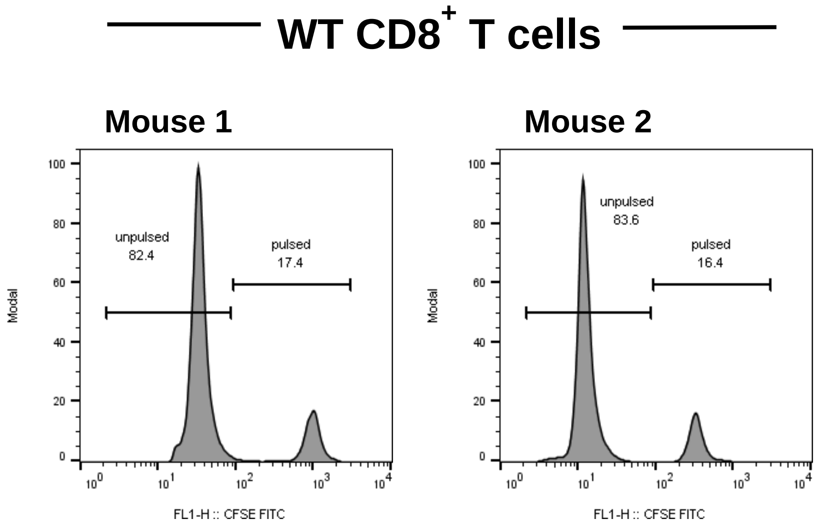

Supplementary Figure 7: Antigen-specific killing by WT CD8$^+$ T cells *in vivo.* Spleen cells from OT-I mice were adoptively transferred into C57BL/6J mice (n=2 mice) followed by s.c. vaccination with OVA peptide and anti-CD40. Imiquimod cream was applied on the vaccination site. In addition, mice received 100,000 IU rhIL-2 by i.p. Three days after vaccination, mice were injected with a 1:1 mix of target cells. Target cells were prepared using splenocytes from C57BL/6 mice either loaded with 1 µg/ml peptide and labeled with 5 µmol/L CFSE or unloaded and labeled with 0.5 µmol/L CFSE. 8 h later, splenocytes from the recipients were analyzed by flow cytometry to assess peptide-specific killing of the CFSE^{hi} labeled target cells.

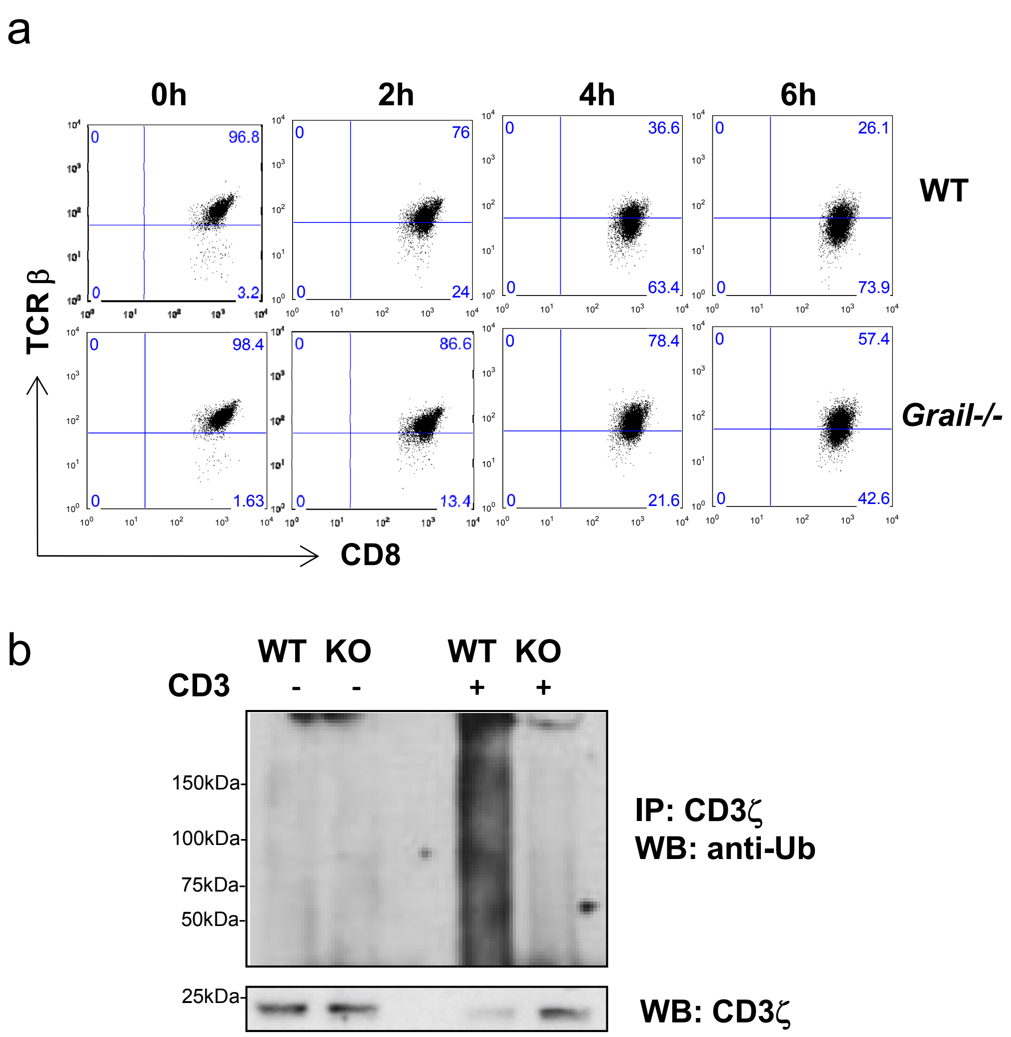

Supplementary Figure 8: Grail controls TCR/CD3 expression in $CD8^+$ T cells. (**a**) Splenic T cells from WT and *Grail-/-* OT-I mice were stimulated with OVA peptide and the expression of TCRβ on $CD8^+$ T cells was determined by flow cytometry. The results shown are representative of two independent experiments. (**b**) CD8 T cells from WT and *Grail-/-* mice were untreated or activated with anti-CD3 for 6 hours. MG-132 was added to all samples for 6 hours. The cell lysates were ip using anti-CD3ζ antibodies. The blot was probed with anti-Ub antibodies, and re-probed with anti-CD3ζ-HRP. The results shown are a representative of three independent experiments.

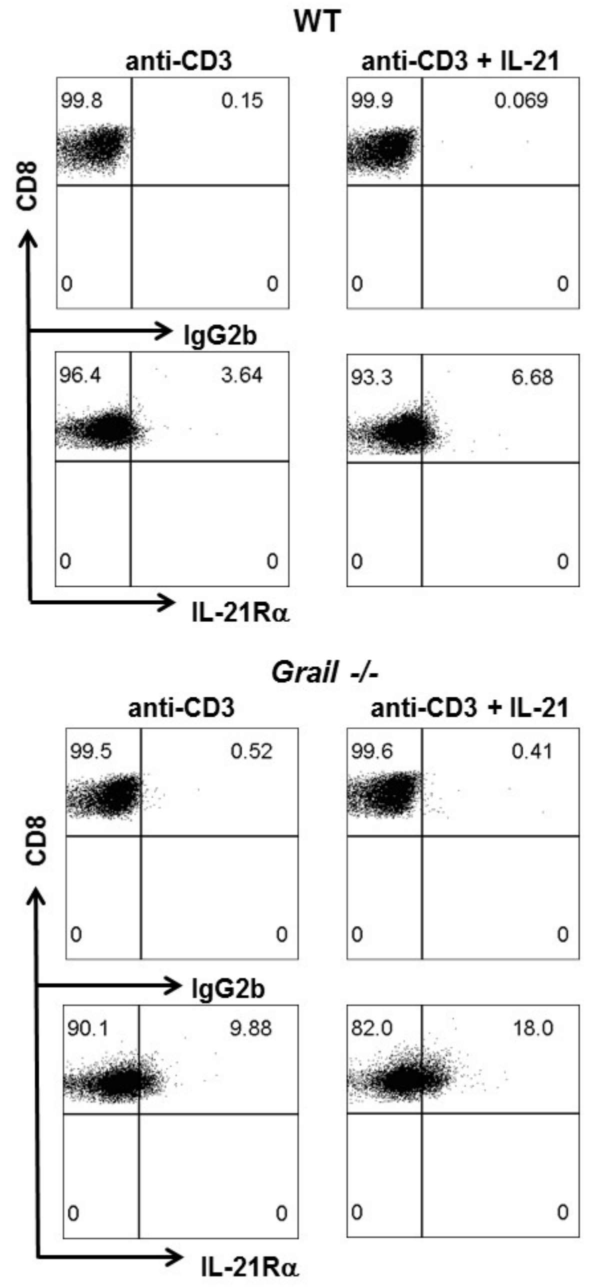

Supplementary Figure 9: Specificity of IL-21R staining. Naïve WT and *Grail-/-* $CD8^+$ T cells were stimulated with anti-CD3 in the presence or absence of IL-21 for 3 days. IL-21R expression on $CD8^+$ T cells was assessed flow cytometry staining with isotype control (rat IgG2b kappa) and IL-21Rα antibodies. Numbers in dot plot quadrants represent the percentages of each subset.

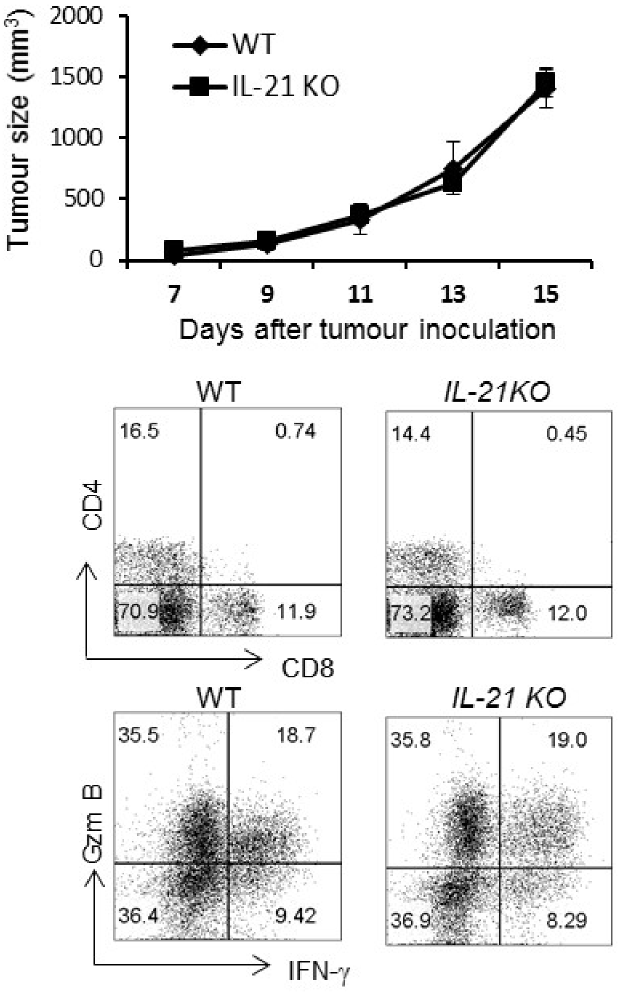

Supplementary Figure 10: Absence of IL-21 does not influence tumour growth or level of T cell infiltration and functionality. WT and IL-21KO mice were inoculated with EG-7 tumour cells. Tumour size was measured and calculated as in Figure 1**a**. TILs were stained and analyzed as described in Figure 1. All experiments were independently performed twice with 7 mice per group.

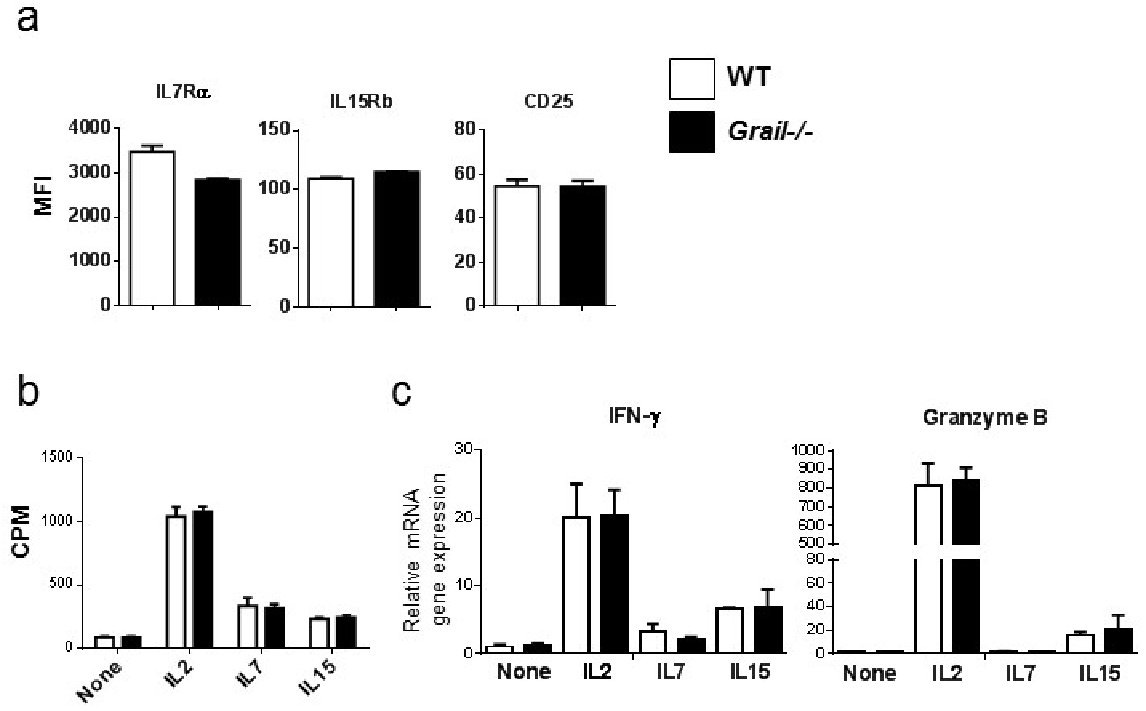

Supplementary Figure 11: Normal IL-2, IL-7 and IL-15 signaling in naïve *Grail-/-* CD8$^+$ T cells. (**a**) Untreated WT and *Grail-/-* splenocytes were stained with anti-IL7Rα, IL15Rb, CD25, CD8, and CD44 and analyzed in CD8$^+$CD44^{neg} gate. (**b**) Naïve WT and *Grail-/-* CD8$^+$ T cells were activated with anti-CD3 alone or together with IL-2, IL-7 or IL-15 treatment. Proliferation was assayed 72 hrs after treatment by adding [^{3}H]-thymidine to the culture for the last 8 hrs. (**c**) The mRNA gene expression of IFN-γ and Granzyme B 24 hrs after activation was analyzed by RT-PCR. All experiments were independently performed twice. MFI: mean fluorescence intensity, CPM: count per minute.

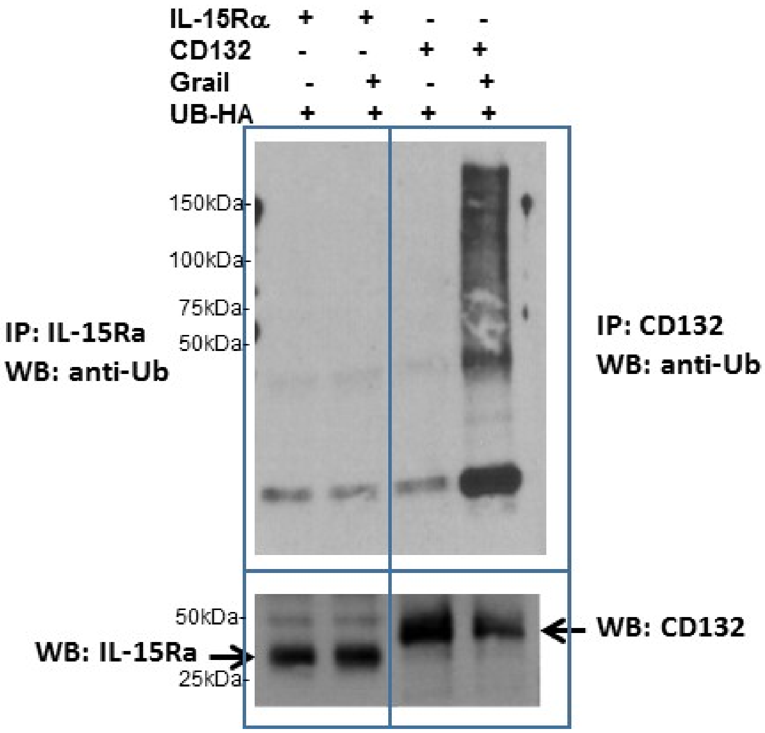

Supplementary Figure 12: Grail controls common gamma chain ubiquitination but not IL-15Rα. 293T cells were transfected with vectors encoding CD132 or IL-15Rα, HA-Ub, and Grail. The lysates were subjected to immunoprecipitation (i.p.) using anti-IL-15Rα or anti-CD132 antibodies. The blots were probed with anti-HA-HRP and re-probed with anti-IL-15Rα or anti-CD132. All experiments were independently performed twice.

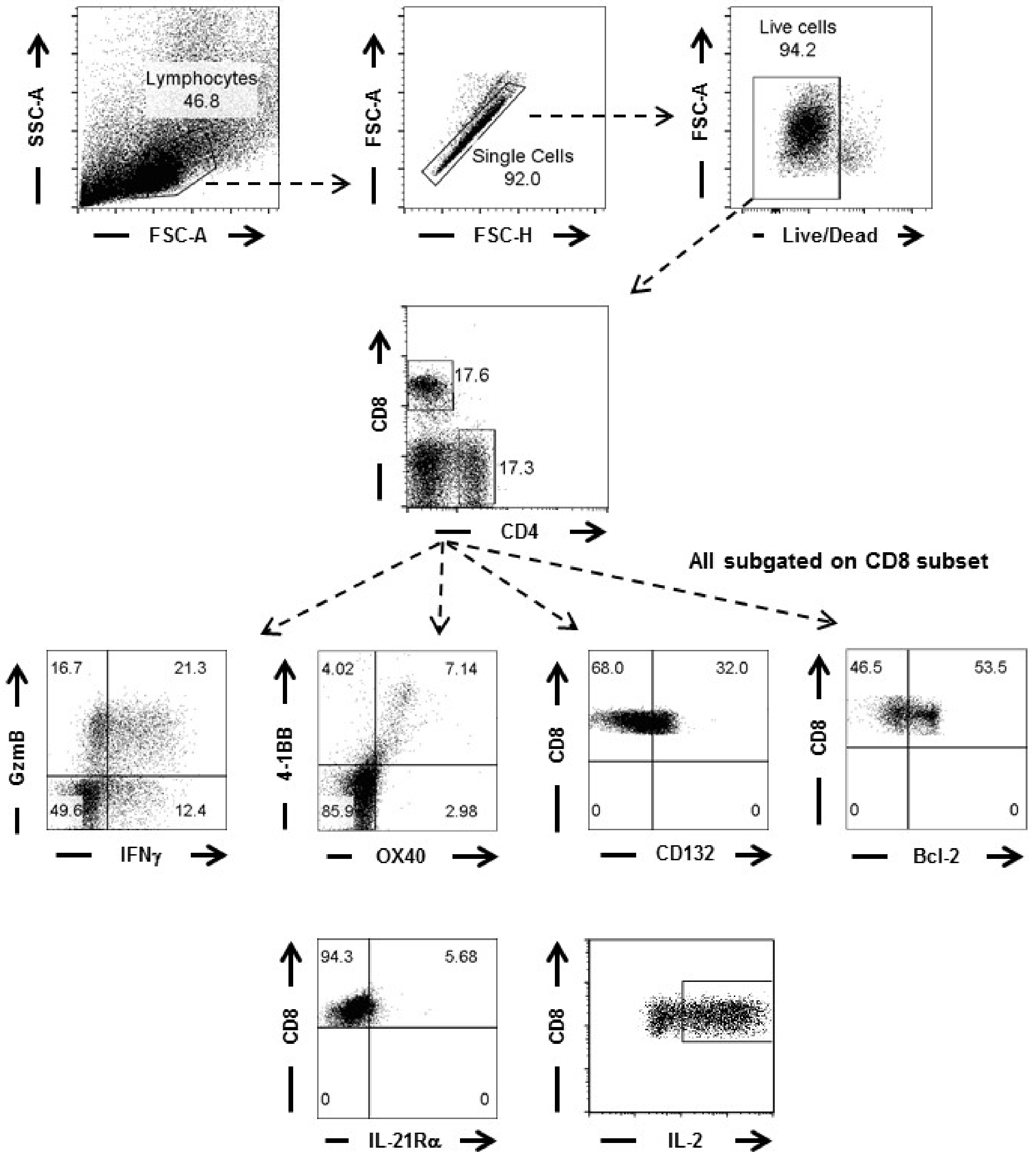

Supplementary Figure 13: Gating strategy for CD8+ T cell phenotyping. For all *in vivo* and *in vitro* assays, CD8+ T cell phenotyping was performed using the above flow cytometry gating logic.

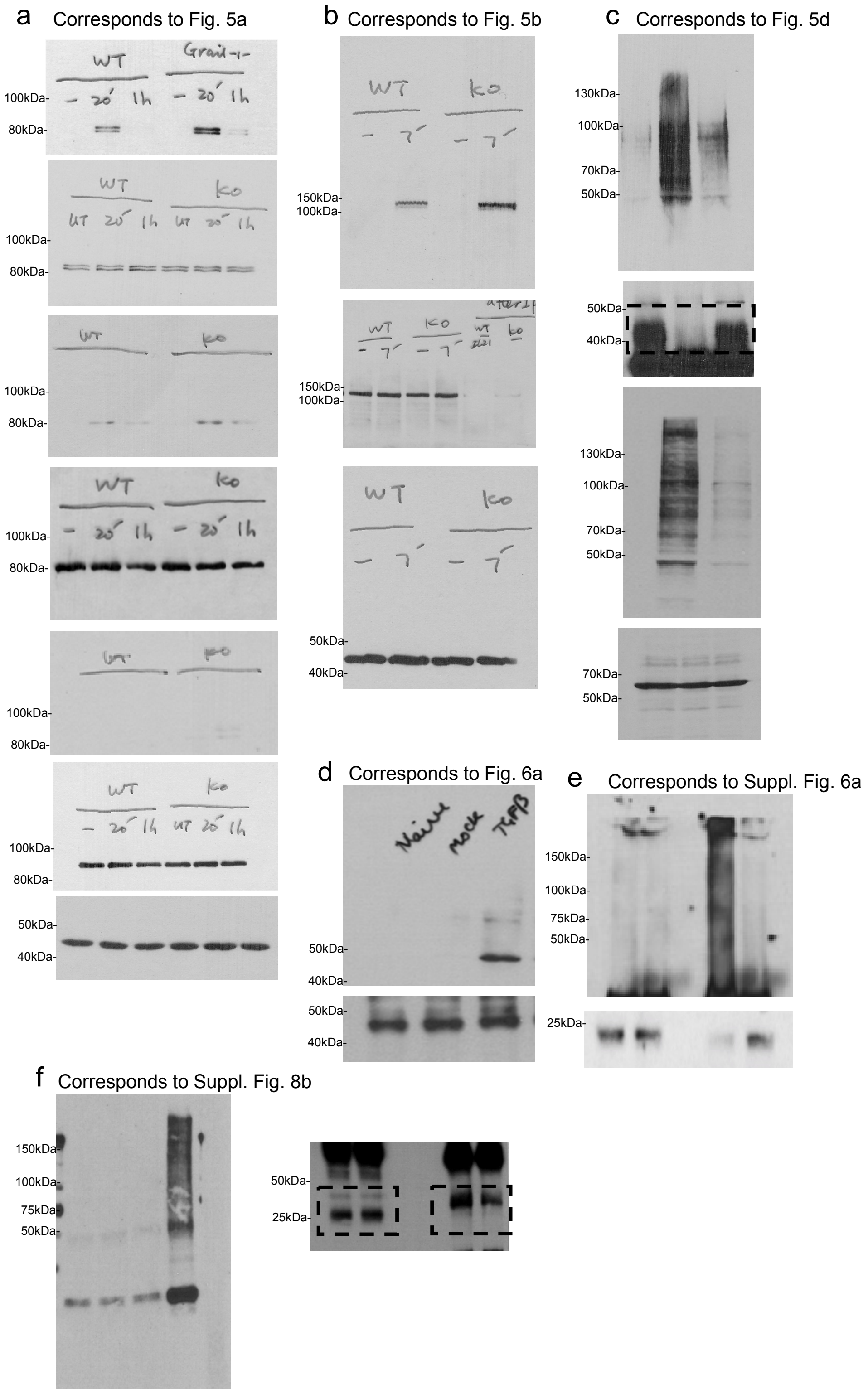

Supplementary Figure 14: Uncropped gel images for western blot and ubiquitination assays. (a-f) shows the uncropped gel images along with the associated main or supplementary figure reference.
